# Antimicrobial-resistant Invasive *Escherichia coli*, Spain

**DOI:** 10.3201/eid1104.040699

**Published:** 2005-04

**Authors:** Jesús Oteo, Edurne Lázaro, Francisco J. de Abajo, Fernando Baquero, José Campos, Spanish members of EARSS

**Affiliations:** * Instituto de Salud Carlos III, Madrid, Spain;; † Agencia Española del Medicamento;; ‡ Hospital Ramón y Cajal, Madrid, Spain

**Keywords:** invasive pathogens, *Escherichia coli*, antimicrobial susceptibility, Spain, research

## Abstract

To address the public health problem of antimicrobial resistance, the European Union founded the European Antimicrobial Resistance Surveillance System. A network of 32 Spanish hospitals, serving ≈9.6 million persons, submitted antimicrobial-susceptibility data on 7,098 invasive *Escherichia coli* species (2001–2003). Resistance to ampicillin, cotrimoxazole, ciprofloxacin, gentamicin, and tobramycin was found at rates of 59.9%, 32.6%, 19.3%, 6.8%, and 5.3%, respectively. Resistance to multiple drugs increased from 13.8% in 2001 to 20.6% in 2003 (p <0.0001). Antimicrobial consumption data were obtained from the Spanish National Health System. In spite of decreased cephalosporin and β-lactam use, overall extended-spectrum β-lactamase production increased from 1.6% (2001) to 4.1% (2003) (p <0.0001), mainly due to the rising prevalence of cefotaximases. Resistance to ciprofloxacin significantly increased, mostly in community-onset infections, which coincided with a rise in community quinolone use. Cotrimoxazole resistance remained stable at ≈30%, even though its use was dramatically reduced.

## Abstract 2

Antimicrobial resistance, particularly to fluoroquinolones and third-generation cephalosporins, is increasing in *E. coli* in Spain.

Antimicrobial resistance is a well-known clinical and public health problem (*1*). For example, in the United States in 2002, resistance to ampicillin and ciprofloxacin among 5,192 *Escherichia coli* blood isolates was 47.8% and 13.3%, respectively (*2*). The World Health Organization (WHO), the European Commission, and the U.S. Centers for Disease Control and Prevention (CDC) have recognized the importance of studying the emergence and determinants of resistance as well as the need for control strategies (*1**,**3**,**4*).

The European Antimicrobial Resistance Surveillance System (EARSS) is an international network of national surveillance systems that attempts to collect reliable and comparable antimicrobial resistance data of invasive pathogens. The International Network for the Study and Prevention of Emerging Antimicrobial Resistance has similar goals (*3*). The purpose of EARSS is to document variations in antimicrobial resistance over time and space to provide the basis for developing prevention programs, making policy decisions, and assessing the effectiveness of both.

*E. coli* is one of the main causes of both nosocomial and community-acquired infections in humans (*5*) and one of the microorganisms most frequently isolated from blood (*2**,**6**–**8*). Pathogenic isolates of *E. coli* have a relatively large potential for developing resistance (*2**,**5**,**7**,**9*). In recent years, fluoroquinolone resistance has increased in some countries (*2**,**10**,**11*), CTX-M-type extended-spectrum β-lactamase (ESBL) dissemination has been described (*12**,**13*), and reports of multidrug resistance are not infrequent (*9**,**14**,**15*).

Among western countries, Spain has one of the highest rates of antimicrobial consumption (*16**,**17*) and antimicrobial resistance (*15*). The goal of this prospective study was to describe and analyze the evolution of antimicrobial resistance in comparison to antimicrobial use. Using 7,098 blood or cerebrospinal fluid (CSF) isolates of *E. coli* collected by Spanish hospitals participating in the EARSS network from 2001 to 2003, we found that antimicrobial resistance, particularly to fluoroquinolones and third-generation cephalosporins, was increasing in *E. coli*.

## Materials and Methods

### Selection of Participating Hospitals

To fulfill the goal of obtaining representative data, participating hospitals were chosen to meet the following criteria: 1) coverage of at least 20% of the Spanish population, 2) different areas of the country covered, and 3) different kinds of hospitals (size and category) represented. The official catalog of all available Spanish hospitals, as published by Spanish Ministry of Health, was used to randomly select hospitals involved in this surveillance system; 3 hospitals refused to participate were replaced by 3 other hospitals of similar characteristics.

### Strains Studied

All clinical isolates of *E. coli* obtained from blood and CSF samples in microbiology laboratories of Spanish hospitals that participated in EARSS from 2001 to 2003 were included. Only the first invasive isolate per patient was reported. Invasive infection was defined as infection with an *E. coli* isolate from blood or CSF. Nosocomial infections were defined as infections acquired at least 48 h after hospital admission. Patients with community-acquired infections were those who had positive cultures by *E. coli* at the time of or within 48 h of hospitalization.

### Data Collection

A questionnaire concerning hospital characteristics (coverage, hospital type, number of beds, number of patients admitted per year, hospital departments), methods of antimicrobial susceptibility study, and interpretation criteria was completed by each participating center. One isolate record form was completed for each patient. This form included personal patient data (code, age, sex), hospital and departmental data, and antimicrobial susceptibility data.

Participating hospitals sent prospectively standardized results to the Ministry of Health, where results were analyzed and validated by using the laboratory-based WHONET 5 program (WHO Collaborating Center for the Surveillance of Antibiotic Resistance). A medical microbiologist carefully reviewed all records.

Only the first isolate per patient and year was included. Discrepancies and atypical results were resolved by telephone inquiry, and the corresponding database records were updated if necessary. At the end of each year, an annual report of all data stored in the central database was sent to each participating laboratory to avoid possible discrepancies.

### Antimicrobial Susceptibility Studies

The protocol for *E. coli* susceptibility testing included the following antimicrobial agents: ampicillin, aminoglycosides (gentamicin and tobramycin), fluoroquinolones (ciprofloxacin), and third-generation cephalosporins (cefotaxime and ceftazidime). Data on antimicrobial susceptibility to additional antimicrobial agents were also considered when this information was available for at least 5,900 isolates. For this reason, the number of strains studied for each antimicrobial agent in some cases was not the same as the total number of strains.

Each laboratory identified strains and tested their susceptibility according to standard microbiologic procedures. In 29 laboratories, identification and antimicrobial susceptibility tests were performed by using the following commercial microdilution systems: 14 used MicroScan (Dade-Behring, Deerfield, Illinois, USA); 8, Wider (Fco. Soria Melguizo S.A., Madrid, Spain); 5, Vitek (bioMérieux, Marcy l'Etoile, France); and 2, Sensititre (Radiometer/Copenhagen Company, Denmark). The 3 remaining laboratories used the disc-plate diffusion method combined with E test strips (AB-Biodisk, Solna, Sweden). Results were scored as susceptible, intermediate, or resistant according to criteria established by the National Committee for Clinical Laboratory Standards (NCCLS, now the Clinical and Laboratory Standards Institute) (*18*).

Based on NCCLS criteria, a consensus guideline for detecting ESBL production was recommended by EARSS to all participants (*18*). ESBL producers were considered resistant to both cefotaxime and ceftazidime independent of their MIC in accordance with NCCLS criteria (*18*). Multidrug resistance was defined as resistance to ≥3 of the antimicrobial agents tested.

### Quality Control

To assess the comparability of susceptibility test results, a quality assurance exercise was performed yearly among the 32 participating laboratories. The U.K. National External Quality Assessment Scheme designed the quality controls. Altogether, 24 well-characterized control invasive strains, including 6 *E. coli* strains with different resistance phenotypes, were tested. All these external quality control strains were recommended to be included in the regular internal quality control procedures performed by each laboratory. Data on susceptibility to ampicillin, ciprofloxacin, gentamicin, cefotaxime, and ceftazidime were required. In addition, each laboratory completed a questionnaire concerning the methods used for determining susceptibility and applying interpretation criteria.

### Community Antimicrobial Use

The Ministry of Health and Consumer Affairs maintains a drug database of retail pharmacy sales of all medicines acquired with National Health System prescriptions, covering nearly 100% of the Spanish population (*17**,**19*). These data reflect the outpatient antimicrobial use in Spain. This database was used to gather information on sales for the period 1998–2003. The information was tabulated, and the number of units sold was converted into defined daily doses (DDD) of the active drug ingredients in accordance with WHO guidelines (*20*). We then calculated the number of DDD per 1,000 inhabitants per day for each of the active drug ingredients. This information was not available in relation to patient age.

### Statistical Analyses

Differences in the prevalence of antimicrobial resistance between different groups were assessed by Fisher exact test. Association was determined by calculation of the odds ratio (OR) with 95% confidence intervals (CI). The null hypothesis was rejected for values of p < 0.05. Statistical analyses were performed with EpiInfo version 6.04 software (CDC, Atlanta, GA, USA).

## Results

### Characteristics of Participating Laboratories

From 2001 to 2003, a total of 32 laboratories reported data on invasive *E. coli* isolates. The estimated average coverage of the Spanish population was 23%, which corresponds to ≈9.5 million persons. The median annual numbers of hospital beds and patients admitted were ≈14,500 and 550,000, respectively. Four hospitals (12.5%) had >1,000 beds, 8 (25%) had 500–1,000 beds, 15 (46.9%) had 250–499, and 5 (15.6%) had <250. Twelve (37.5%) were university or tertiary-care hospitals, and 20 (62.5%) were general or secondary-care hospitals.

### Quality Control Results

Among participating laboratories, the overall concordance of susceptibility to ampicillin, gentamicin, and ciprofloxacin in the 6 *E. coli* control strains was 100%, 89%–100%, and 92%–100%, respectively. ESBL production was detected by 85.2%–97% of the laboratories. The participating laboratories used NCCLS-recommended procedures for ESBL detection (*18*).

In the few cases of disagreement between the expected quality control results and the actual performance of individual laboratories, individual cases were analyzed and discussed with participants. Measures to improve laboratory procedures were proposed when necessary, including the dispatch of isolates to the Spanish *E. coli* reference laboratory (352 [4.9%] strains submitted during the study period).

### Patient Data

Data on 7,098 isolates of *E. coli*, corresponding to the same number of patients, were reported, including 3,484 (49.1%) male patients, 3,581 (50.5%) female patients, and 33 of unknown sex. All isolates were collected from blood except 9 from CSF. Of the total number of isolates, 309 (4.4%) were from children ≤14 years of age, 2,145 (30.2%) were from patients ≥15 and ≤64 years of age, and 4,644 (65.4%) were from patients >64 years of age. A total of 3,339 (47.3%) isolates were implicated in nosocomial infections (1,465 from internal medicine, 442 from surgery, 309 from pediatrics, 290 from intensive care units, 81 from infectious diseases, 75 from obstetrics and gynecology, and 677 from other departments), and 3,735 (52.6%) isolates were implicated in community-acquired infections; in 24 cases this information was missing.

### Antimicrobial Susceptibility

The antimicrobial susceptibility of the *E. coli* isolates studied is shown in Table 1. In the *E. coli* isolates, resistance to ampicillin, cotrimoxazole, ciprofloxacin, gentamicin, and tobramycin was found at rates of 59.9%, 32.6%, 19.3%, 6.8%, and 5.3%, respectively. Of the 7,098 isolates tested for cefotaxime, 234 isolates (3.3%) were nonsusceptible, including 19 (0.3%) intermediate and 215 (3%) resistant. ESBL producers totaled 204 (2.9% of all strains tested for cefotaxime) isolates. Ceftazidime susceptibility data were available for 5,960 isolates. Of these, 209 (3.5%) were nonsusceptible, including 10 (0.2%) intermediate and 199 (3.3%) resistant.

**Table 1:** Antimicrobial susceptibility in invasive isolates of *Escherichia coli*, Spain, 2001–2003*

Antimicrobial agent	N	S (%)	I (%)	R (%)
Ampicillin	7,098	2,884 (40.6)	34 (0.5)	4,180 (59.9)
Cefotaxime	7,098	6,830 (96.7)	19 (0.3)	215 (3.0)†
Ceftazidime	5,960	5,751 (96.5)	10 (0.2)	199 (3.3)‡
Ciprofloxacin	7,078	5,673 (80.1)	33 (0.6)	1,372 (19.3)
Gentamicin	7,074	6,558 (92.7)	34 (0.5)	482 (6.8)
Cotrimoxazole	6,597	4,432 (67.2)	11 (0.2)	2,154 (32.6)
Tobramycin	6,135	5,688 (92.7)	122 (2.0)	325 (5.3)

*S, susceptible; I, intermediate; R, resistant. †204 extended-spectrum β-lactamase (ESBL) producers. ‡185 ESBL producers.

Among the 185 *E. coli* ESBL producers in which susceptibility data to both cefotaxime and ceftazidime were reported, nonsusceptibility to cefotaxime according to MIC data was found in 113 (61.1%) cases, while nonsusceptibility to ceftazidime was reported in 68 cases (36.8%). Resistance figures to other antimicrobial agents were as follows: imipenem, 0% of 4,504 isolates tested; amikacin, 0.3% of 4,484 isolates tested; and amoxicillin/clavulanic acid, 6% intermediate and 4.5% resistant of 3,023 isolates tested.

The prevalence of antimicrobial resistance was higher in male patients than in female patients (Table 2), particularly for ciprofloxacin, gentamicin, and cotrimoxazole. Nosocomial isolates were significantly more resistant to ampicillin, ciprofloxacin, cotrimoxazole, gentamicin, and cefotaxime than community-acquired isolates (Table 3). Of the 204 ESBL producers, 66 (32.4%) were implicated in community-onset infections. Resistance to ciprofloxacin was higher in nosocomial isolates from hospitals with >500 beds than in those from hospitals with ≤500 beds, 24.6% vs. 21.3% (p = 0.02, OR 1.2, 95% CI 1.02–1.42). No statistical differences were found in the resistance figures to other antimicrobial agents according to hospital size. In general, antimicrobial resistance did not vary in relation to hospital departments; however, resistance to gentamicin was more prevalent in intensive care units than in internal medicine, 10.5% vs. 6.8% (p = 0.04, OR 1.57, 95% CI 1.02–2.40).

**Table 2:** Prevalence of antimicrobial resistance in invasive isolates of *Escherichia coli* in relation to patient sex*

Antimicrobial agent	Male		Female			
	N	R% (n)	N	R% (n)	p	OR (CI 95%)
Ampicillin	3,484	58.8 (2,049)	3,581	56.5 (2,023)	0.05	1.10 (1.00–1.21)
Ciprofloxacin	3,478	22.8 (793)	3,570	16.3 (582)	<0.0001	1.52 (1.34–1.71)
Cotrimoxazole	3,240	32.9 (1,066)	3,329	29.5 (982)	0.002	1.17 (1.05–1.30)
Gentamicin	3,474	8.8 (306)	3,570	5.1 (182)	<0.0001	1.80 (1.48–2.18)
Cefotaxime	3,468	3.5 (121)†	3,566	2.5 (89)†	0.01	1.41 (1.06–1.88)

*R%, percent resistance; OR, odds ratio; CI, confidence interval. †Include isolates with intermediate susceptibility and resistance.

**Table 3:** Prevalence of antimicrobial resistance in nosocomial and community-acquired invasive isolates of Escherichia coli*

Antimicrobial agent	Nosocomial		Community-acquired			
	N	R% (n)	N	R% (n)	p	OR (CI 95%)
Ampicillin	3,337	61 (2,036)	3,734	54.6 (2,039)	<0.0001	1.64 (1.49–1.81)
Ciprofloxacin	3,325	22.6 (751)	3,730	16.7 (623)	<0.0001	1.46 (1.29–1.64)
Cotrimoxazole	3,098	34.3 (1,063)	3,484	28.2 (982)	<0.0001	1.33 (1.20–1.48)
Gentamicin	3,328	8.8 (293)	3,721	5.2 (193)	<0.0001	1.76 (1.46–2.14)
Cefotaxime	3,315	4.4 (146)†	3,724	1.9 (71)†	<0.0001	2.37 (1.76–3.19)
Multiresistance	2,414	19.3 (466)	2,586	13.1 (339)	<0.0001	1.59 (1.36–1.85)

*R%, percent resistance; OR, odds ratio; CI, confidence interval. †Includes isolates with intermediate susceptibility and resistance.

Isolates from children ≤14 years of age were significantly more resistant to ampicillin than those from patients >14 years of age, 63% vs. 57.4% (p = 0.047, OR 1.27, 95% CI 1–1.62). In contrast, ciprofloxacin resistance was less prevalent in children than in adults, 8.8% vs. 20% (p < 0.001, OR 0.38, 95% CI 0.25–0.58). In the other antimicrobial agents tested, no differences relating to patient age were apparent. Among the 27 ciprofloxacin resistance isolates from children, 3 (11.1%) were also ESBL producers.

Resistance to cotrimoxazole, ciprofloxacin, and gentamicin was more prevalent in ampicillin-resistant (46.7%, 27.7%, and 10.8%, respectively) strains than in ampicillin-susceptible strains (9.9%, 8.5%, and 1.8%, respectively) (p < 0.001). Also, *E. coli* ESBL-producing strains were significantly more resistant to other non–β-lactam antimicrobial agents than nonproducing strains, as was the case for ciprofloxacin (57.4% vs. 18.4%; p < 0.001), cotrimoxazole (56.8% vs. 30.3%; p < 0.001), and gentamicin (22.5% vs. 6.5%; p < 0.001). Of the 1,372 ciprofloxacin-resistant isolates, 113 (8.2%) were also ESBL producers. In contrast, of 5,673 ciprofloxacin susceptible isolates, only 91 (1.6%) were ESBL producers (p < 0.001, OR 5.59, 95% CI 4.21–7.42).

Of the 5,018 (70.7%) strains tested for simultaneous susceptibility to ampicillin, ciprofloxacin, gentamicin, cotrimoxazole, cefotaxime and ceftazidime, multidrug resistance was present in 863 (17.2%) isolates. The most prevalent phenotypes included resistance to ampicillin, cotrimoxazole, and ciprofloxacin, which was detected in 382 isolates (44.3% of multidrug-resistant strains and 7.6% of strains overall) and resistance to ampicillin, cotrimoxazole, ciprofloxacin, and gentamicin, detected in 151 strains (17.5% of multidrug-resistant strains and 3% of strains overall).

### Trends in Antimicrobial Resistance

**Figure 1:**
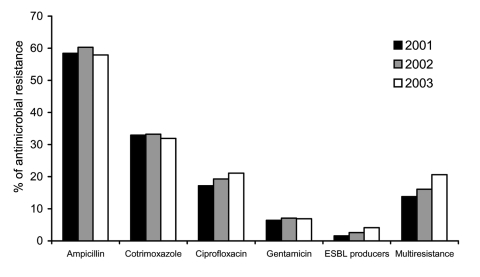
Annual evolution of antimicrobial resistance in invasive *Escherichia coli* isolated by Spanish laboratories participating in European Antimicrobial Resistance Surveillance System, 2001–2003.

**Figure 2:**
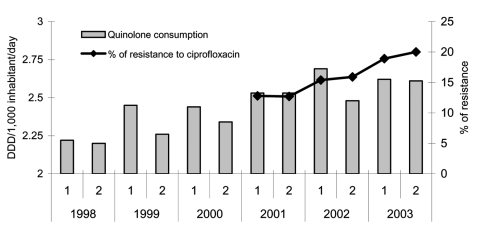
Evolution of community quinolone consumption and prevalence of resistance to ciprofloxacin in invasive community-acquired *Escherichia coli* infections (European Antimicrobial Resistance Surveillance System–Spain 2001–2003). DDD, defined daily doses. 1, January–June; 2, July–December.

Ampicillin and cotrimoxazole resistance did not significantly vary over the study period, from 58.4% (2001) to 57.9% (2003) and from 32.9% (2001) to 31.9% (2003), respectively (Figure 1). However, resistance to ciprofloxacin increased from 17.2% in 2001 to 21.1% in 2003 (3.9% change) (p < 0.001, OR 1.29, 95% CI 1.11–1.50) (Figure 2).

**Figure 3:**
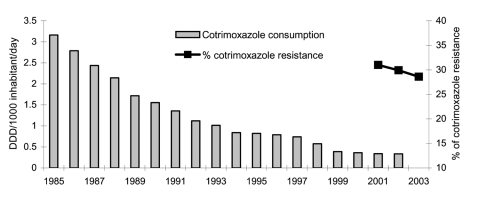
Annual evolution of community cotrimoxazole consumption and prevalence of resistance to cotrimoxazole in invasive community-acquired *Escherichia coli* infections, European Antimicrobial Resistance Surveillance System, Spain, 2001–2003. DDD, defined daily dose.

The prevalence of ciprofloxacin resistance in community-acquired isolates increased from 13.3% in 2001 to 19.3% in 2003 (6% change) (p = 0.0002, OR 1.56, 95% CI 1.22–1.98), a higher increase than that observed for all strains. Figures 2 and 3 show the evolution of community quinolone and cotrimoxazole use compared with resistance to ciprofloxacin and cotrimoxazole, respectively, in invasive community-acquired *E. coli* infections. In the first case (Figure 2), both parameters increased, but cotrimoxazole use was strongly reduced from 1965 to 2003, while resistance figures remained near 30% (2001–2003) (Figure 3).

The global rates of invasive *E. coli* ESBL producers increased from 1.6% (2001) to 4.1% (2003) (2.5% change) (p < 0.0001, OR 2.70, 95% CI 1.77–4.15) (Figure 1). Community-acquired ESBL producers increased from 0.4% (2001) to 1.5% (2003) (1.1% change) (p < 0.001, OR 3.74, 95% CI 1.68–8.67).

Regarding susceptibility to third-generation cephalosporins, the number of strains nonsusceptible to cefotaxime (MIC >8 µg/mL) but susceptible to ceftazidime (MIC ≤8 µg/mL), increased from 26.5% in 2001 to 39.8% in 2003 (13.3% change) (p < 0.0001, OR 1.83, 95% CI 1.59–2.12). The prevalence of multidrug resistance among isolates tested for ampicillin, ciprofloxacin, gentamicin, cotrimoxazole, cefotaxime, and ceftazidime was 13.8% in 2001, 16.1% in 2002, and 20.6% in 2003 (p < 0.0001, OR 1.62, CI 95% 1.33–1.97) (Figure 1).

### Antimicrobial Use

Total β-lactam use decreased from 13.34 DDD/1,000 inhabitants/day in 1998 to 11.44 DDD/1,000 inhabitants/day in 2003 (14.5% change). Consumption of broad-spectrum penicillins and cephalosporins decreased from 6.02 to 4.52 DDD/1,000 inhabitants/day (24.9% change) and from 2.65 to 2.20 DDD/1,000 inhabitants/day (17% change), respectively. In contrast, the use of amoxicillin/clavulanate (4.67 DDD/1,000 inhabitants/day in 1998 to 6.54 DDD/1,000 inhabitants/day in 2003) and quinolones (1.96 DDD/1,000 inhabitants/day in 1998 to 2.69 DDD/1,000 inhabitants/day in 2003) increased by 40% and 37.2%, respectively. Ciprofloxacin use remained stable; levofloxacin and moxifloxacin use increased. From 1998 to 2003, cotrimoxazole consumption was very low and decreasing. However, when analyzed from 1985, cotrimoxazole use decreased by 89.4%, from 3.2 DDD/1,000 inhabitants/day to 0.34 DDD/1,000 inhabitants/day (Figure 3).

## Discussion

Epidemiologic surveillance of antimicrobial resistance is indispensable for empirically treating infections, implementing resistance control measures, and preventing the spread of antimicrobial-resistant microorganisms (*21*). The EARSS network, which includes >700 laboratories, is the official European network of national surveillance systems. It aims to collect comparable and reliable antimicrobial resistance data, with susceptibility data provided by each microbiology laboratory according to standard methods, mainly based on NCCLS rules.

This European network has some important characteristics as a surveillance system for resistance to antimicrobial agents (*22*). These characteristics include the following: 1) aggregation of data by each individual country and overall European countries, 2) rapid analysis and diffusion of data, 3) early detection systems for antimicrobial resistance in pathogens of clinical and public health relevance, and 4) basic decision support for public health.

Use of the information generated by the primary clinical laboratory has several disadvantages, namely, the possible variability in the antimicrobial agents assayed, the study methods used, and the interpretative criteria employed. In our experience, however, most laboratories used NCCLS-recommended methods. Previous validation of antimicrobial susceptibility results from 22 European countries, including Spain, has been performed by EARSS researchers (*23*). In addition, cross-validation of routine data gathering and centralized surveys has been implemented previously (*24*).

In this study, *E. coli* ESBL producers were infrequent (2.9%) but much higher than the 0.36% found in 1,918 European clinical blood isolates of *E. coli* isolated from 1997 to 1998 (*7*). One of the ESBL producer strains included in the quality control was undetected by 15% of the laboratories; this potential misclassification could lead to underestimates of the prevalence of ESBL isolates in this study. When ceftazidime nonsusceptibility was used as a surrogate marker for ESBL, 1.2% of 71,800 *E. coli* isolated from blood in the United States were nonsusceptible to ceftazidime (*25*).

In addition, 32.4% of ESBL producers were implicated in community-acquired infections. Although no data about possible previous healthcare contact of the persons infected with ESBL in the community were available, the spread of these types of β-lactamases outside hospitals is a matter of great concern.

We found a significant increase in ESBL production in recent years in Spain, which affected both total and community-acquired isolates. In addition, the increased prevalence of isolates showing nonsusceptibility to cefotaxime but susceptibility to ceftazidime (26.5% in 2001 vs. 39.8% in 2003) suggests that ESBL cefotaximases were increasing quickly, as described by other studies (*12**,**13*). In 2003 the first report from the United States appeared; it documented the isolation of *E. coli* isolates producing CTX-M–like ESBL (9 strains from 5 U.S. states) (*26*). The emergence of this ESBL-type has important implications for the detection of ESBL *E. coli* producers in clinical and epidemiologic surveys and emphasizes the need for ESBL screening to include both cefotaxime and ceftazidime.

Fluoroquinolone use has increased in many European countries (*11**,**17*), with Spanish consumption increasing from 1.96 DDD/1,000 inhabitants/day in 1998 to 2.69 DDD/1,000 inhabitants/day in 2003 (37.2%). In comparison with other European countries participating in EARSS that provided susceptibility results for at least 750 *E. coli* invasive isolates in 2003, ciprofloxacin resistance in Spain (21.1%) was among the highest in Europe. This figure is lower than that in Portugal (25.8%) and Italy (25.3%) but higher than percentages in such countries as Germany (15.2%), Belgium (11.6%), Greece (9.9%), Ireland (9.6%), France (9.4%), and the Netherlands (6.8%).

Isolates from children had a relatively high prevalence of ciprofloxacin resistance (8.8%), although ciprofloxacin was not used by children. This resistance could be due to the transmission of resistant isolates between adults and children in families, daycare, or school settings and to the use of fluoroquinolones in poultry populations (*10*).

In a recent survey of 494 U.S. hospitals, the prevalence of ciprofloxacin resistance was 6%; it had increased in 40% of the participant hospitals (*27*). Also, among 286,187 isolates of *E. coli* from urinary tract infections in female outpatients in the United States, ciprofloxacin was the only agent studied that demonstrated a consistent stepwise increase in resistance from 1995 (0.7%) to 2001 (2.5%) (*28*). In our study, a significant increase in ciprofloxacin resistance, principally in community-onset infections, coincided with rising community quinolone use. Association between fluoroquinolone use and quinolone-resistant *E. coli* has been described recently (*29*).

Cotrimoxazole resistance remained stable in this study, ≈30%, and similar to the 27% reported in urinary tract infection isolates in 1993 in Spain (*30*), in spite of the great reduction (89.4%) found in community cotrimoxazole use in the last 18 years. A similar situation was described previously with sulfonamide resistance in the United Kingdom (*31*). In areas with high resistance rates maintained over long periods of time, reduction in antimicrobial pressure may have a slower effect, especially in the presence of multidrug resistance (*32*). This may be due to genetic linkage between resistance mechanisms and, therefore, co-selection by using only 1 antimicrobial agent (*31*), or to the reservoir of molecular resistance mechanisms in species of commensal flora (*33*).

Antimicrobial resistance, principally to ciprofloxacin and gentamicin, varied between the sexes, with isolates from male patients more resistant than those from female patients. Similar trends have been described recently in the United States (*9*) and the Netherlands (*11*). Nosocomial isolates were also more resistant than community-acquired ones, similar to findings from a recent study in South Korea (*34*). In both cases, these data probably reflect the tendency for male patients and hospitalized patients to more frequently have complicated urinary tract infections, the principal source of invasive *E. coli*, which may be associated with more chronic pathologic conditions and more antimicrobial treatments. Possibly the most important determining factor in resistance is use of antimicrobial agents, as described for ciprofloxacin (Figure 2) (*29*).

In our study, multidrug resistance was frequent (17.2%) and increased by 50% during the study period (2001–2003). Multidrug resistance in the United States among 38,835 urinary tract infection isolates was 7.1% in 2000 (*9*). Such multidrug resistance has important implications for the empiric therapy of infections caused by *E. coli* and for the possible co-selection of antimicrobial resistance mediated by multidrug resistance plasmids (*35*), as described above.

Because antimicrobial resistance patterns are continually evolving and *E. coli* invasive isolates undergo progressive antimicrobial resistance, continuously updated data on antimicrobial susceptibility profiles will continue to be essential to ensure the provision of safe and effective empiric therapies. Moreover, results obtained from these surveillance systems must be used to implement prevention programs and policy decisions to prevent emergence and spread of antimicrobial resistance.
